# Supplementary material for: Mixed-methods Evaluation of an Expedited Partner Therapy Take-home Medication Program: Pilot Emergency Department Intervention to Improve Sexual Health Equity
Source: West J Emerg Med. 2023 Aug 25;24(5):993–1004. doi: 10.5811/westjem.59506 (PMC10527844; doi:10.5811/westjem.59506)
Supplement: Supplementary file 3 [file wjem-24-993-s003.pdf]

ED Expedited Partner Therapy (ED Discharge Only)

- EPT Clinical Policy

Documentation

EPT Progress Note

☑ ED Empiric Expedited Partner Therapy (EPT) Note

Partner Treament

EPT Partner Treatment for Chlamydia & Gonorrhea and Trichomonas

Select the check-box for the treatment option(s) on the EPT written prescription. If available, you can choose to offer take-home medication kits (STICK kits) for the partner(s), throu then the partner can take the prescription to their pharmacy.

☑ ED Expedited Partner Therapy (EPT) for Chlamydia & Gonorrhea and Trichomonas

! Routine, External

Status: Normal | Standing | Future

Priority: Routine | Routine

Class: External | External

! Number of partner scripts needed that will be provided to the index patient for Expedited Partner Therapy:

1 | 2 | 3 | 4 | 5 | 6 | 7 | 8 | 9 | 10

Comments: + Add Comments (F6)

Partner Instructions

Patient & Partner Information

☑ Expedited Partner Therapy for Chlamydia, Gonorrhea & Trichomoniasis (Emergency Department) - UMHS (English)

University of Michigan
UH Adult Emergency Department
1500 E Medical Center Dr
Ann Arbor MI 48109

Phone: 734-936-6666
Fax: 734-232-1218

Date: Jun 28, 2021

Name: Expedited Partner Therapy or intended recipient name (if available): ____________

DOB: 01/01/2021 or intended recipient DOB (if available): ____________

☐ **Expedited Partner Therapy (EPT) Not Pregnant Chlamydia & Gonorrhea Treatment**
Cefixime (Suprax) 400 mg capsule: Take 2 capsules (800 mg) by mouth in a single dose (Quantity prescribed: 2 capsules) PLUS — Refill: **0 (Zero)**
Doxycycline hyclate (Vibramycin) 100 mg tablet: Take 1 tablet (100 mg) by mouth twice daily for 7 days (Quantity prescribed: 14 tablets) — Refill: **0 (Zero)**

☐ **Expedited Partner Therapy (EPT) Pregnant Chlamydia & Gonorrhea**
Cefixime (Suprax) 400 mg capsule: Take 2 capsules (800 mg) by mouth in a single dose (Quantity prescribed: 2 capsules) PLUS — Refill: **0 (Zero)**
Azithromycin 500 mg tablet: Take 2 tablets by mouth in a single dose (Quantity prescribed: 2 tablets) — Refill: **0 (Zero)**

☐ **Expedited Partner Therapy (EPT) Trichomoniasis Treatment**
Metronidazole (Flagyl) 500 mg tablet: Take 4 tablets (2 g) by mouth in a single dose (Quantity prescribed: 4 tablets) — Refill: **0 (Zero)**

Signature: ____________

Entered by: Um_Cln, Faculty Physician, MD — NPI:
Authorizing Provider: Um_Cln, Faculty Physician, MD — NPI:
Supervising Provider: — NPI:

Dear Pharmacists,

This is a prescription for Expedited Partner Therapy for Chlamydia & Gonorrhea or Trichomonas..

Michigan Department of Health & Human Services (MDHHS) has authorized healthcare providers to provide antibiotics to treat sexually transmitted infections in the sex par "Expedited Partner Therapy" in place of a name and address and a birth date of January 1 of the current year may be filled based on these regulatory changes.
