# Supplementary figures and images for: Mixed-methods Evaluation of an Expedited Partner Therapy Take-home Medication Program: Pilot Emergency Department Intervention to Improve Sexual Health Equity
Source: West J Emerg Med. 2023 Aug 25;24(5):993–1004. doi: 10.5811/westjem.59506 (PMC10527844; doi:10.5811/westjem.59506)

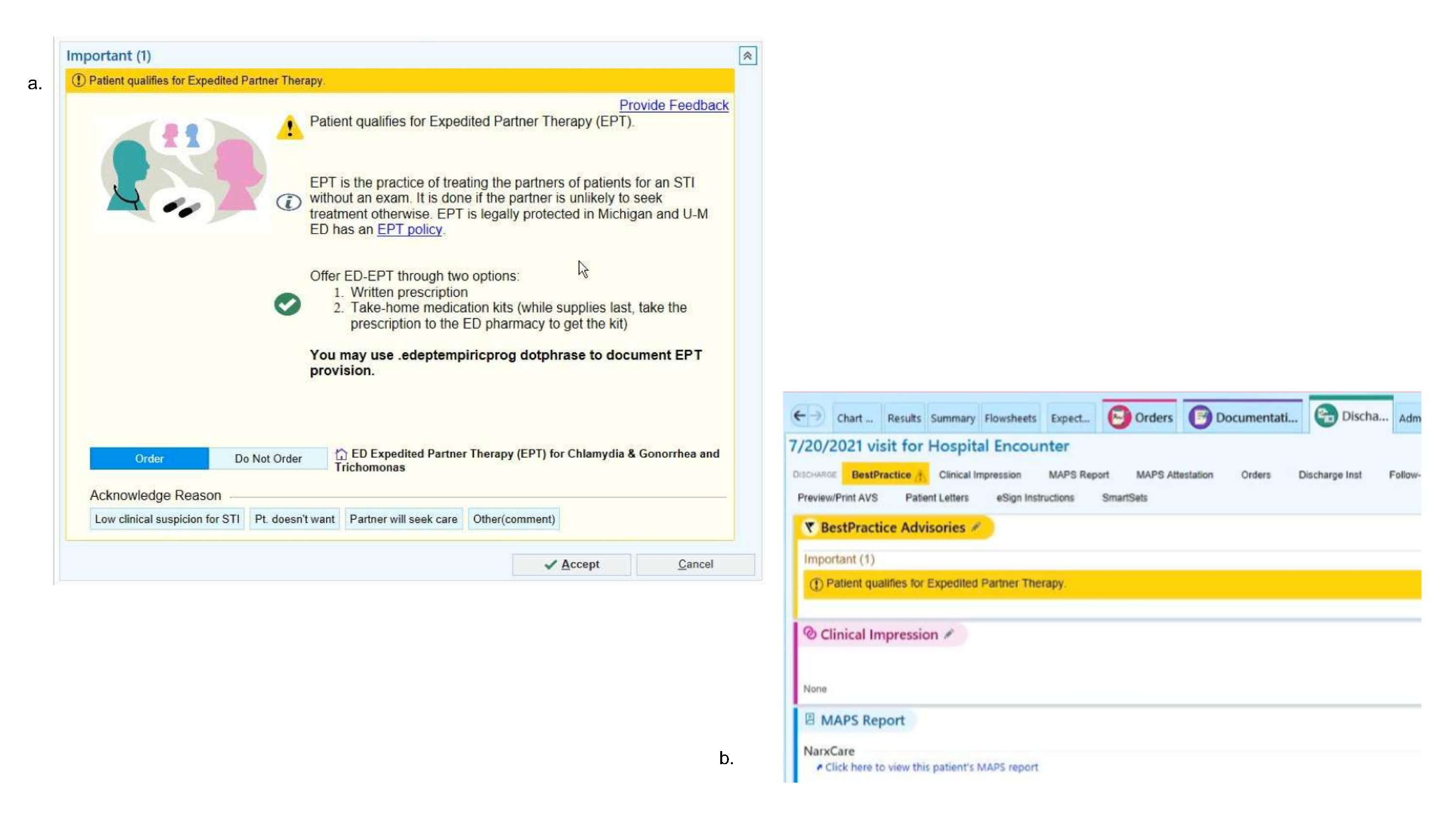

Supplement: Supplementary file 4 [file wjem-24-993-s004.png]
